# Three-dimensional analysis of aligner gaps and thickness distributions, using hard x-ray tomography with micrometer resolution

**DOI:** 10.1117/1.JMI.9.3.031509

**Published:** 2022-06-16

**Authors:** Rémi Ammann, Christine Tanner, Georg Schulz, Bekim Osmani, Prasad Nalabothu, Tino Töpper, Bert Müller

**Affiliations:** a University of Basel, Biomaterials Science Center, Department of Biomedical Engineering, Allschwil, Switzerland; b University of Basel, Biomaterials Science Center, Department of Clinical Research, Basel, Switzerland; c University of Basel, Core Facility Micro- and Nanotomography, Department of Biomedical Engineering, Allschwil, Switzerland; d University Hospital Basel, Department of Oral and Craniomaxillofacial Surgery, Basel, Switzerland; e University of Basel, Center for Dental Medicine, Department of Pediatric Oral Health and Orthodontics, Basel, Switzerland

**Keywords:** optically transparent aligner, advanced high-resolution tomography, aligner gap, aligner thickness distribution, three-dimensional registration, segmentation

## Abstract

**Purpose:**

The morphology of a polymer aligner, designed according to an orthodontic treatment plan, determines clinical outcomes. A fundamental element of orthodontic tooth movement with aligner treatment is the fit of the aligner’s surface to the individual teeth. Gaps between the aligner and teeth do occur because current aligner fabrication is not capable of completely reproducing the complex anatomy of the individual denture. Our study aims at a quantitative three-dimensional assessment of the fit between optically transparent aligners placed on a polymeric model of the upper dental arch for two thermofoil thicknesses at preselected thermoforming temperatures.

**Approach:**

Using an intraoral scan of a subject’s upper dental arch, eight models were printed using a stereolithographic system. A series of eight NaturAligners^®^ was manufactured with a pressure molding process, using thermofoils with thicknesses of 550 and 750  μm and preselected process temperatures between 110°C and 210°C. These aligners placed on the corresponding models were imaged by an advanced micro computed tomography system. The aligners and the models were segmented to extract the gaps and aligners’ local thicknesses as a function of the processing temperature for the two foil thicknesses.

**Results:**

The results indicate that the aligners show a better fit when the foils are processed at higher temperatures. Nevertheless, processing temperatures can be kept below 150°C, as the gain becomes negligible. Thermal processing reduces the average thickness of the aligners to 60% with respect to the planar starting foil. These thickness distributions demonstrate that the aligners are generally thicker on the occlusal surfaces of molars and premolars but thinner around the incisors and buccal as well as on oral surfaces.

**Conclusions:**

Hard x-ray tomography with micrometer resolution is a powerful technique employed to localize the gaps between aligners and teeth, and it also enables film thickness measurements after thermoforming. The thicker film on the occlusal surfaces is most welcome because of aligner abrasion during wear. The NaturAligner^®^ surfaces consist of a 25-μm-thin cellulose layer, and thus the microplastics released via abrasion of less than this thickness are expected to be substantially less critical than for other commercially available, optically transparent aligners.

## 1 Introduction

Orthodontic treatments with optically transparent aligners have seen a constant increase in acceptance in recent years. The low acceptance rates of conventional orthodontic devices in adult populations, due to a perceived lack of attractiveness, as well as workflows becoming easier for practitioners, can explain the rising demand for aligner treatments.^1^^,^^2^ Orthodontic tooth movement is accomplished by employing prolonged pressure on that tooth. The initial movement is rapid, as it occurs within the dental alveolus. The periodontal ligament (PDL), which connects the tooth to the alveolar bone, is stretched on one side and compressed on the other side of the root so that a tension and a compression side can be differentiated.^3^ The disruption of the mechanical balance of PDL and bone leads to the recruitment of osteoclasts and osteoblasts in the vicinity of the tooth.^4^ These specialized cells are responsible for bone resorption on the compression side and bone apposition on the tension side, respectively.^5^ This bone remodeling usually occurs within 40 days after the initial force is applied.^3^

**Fig. 1:**
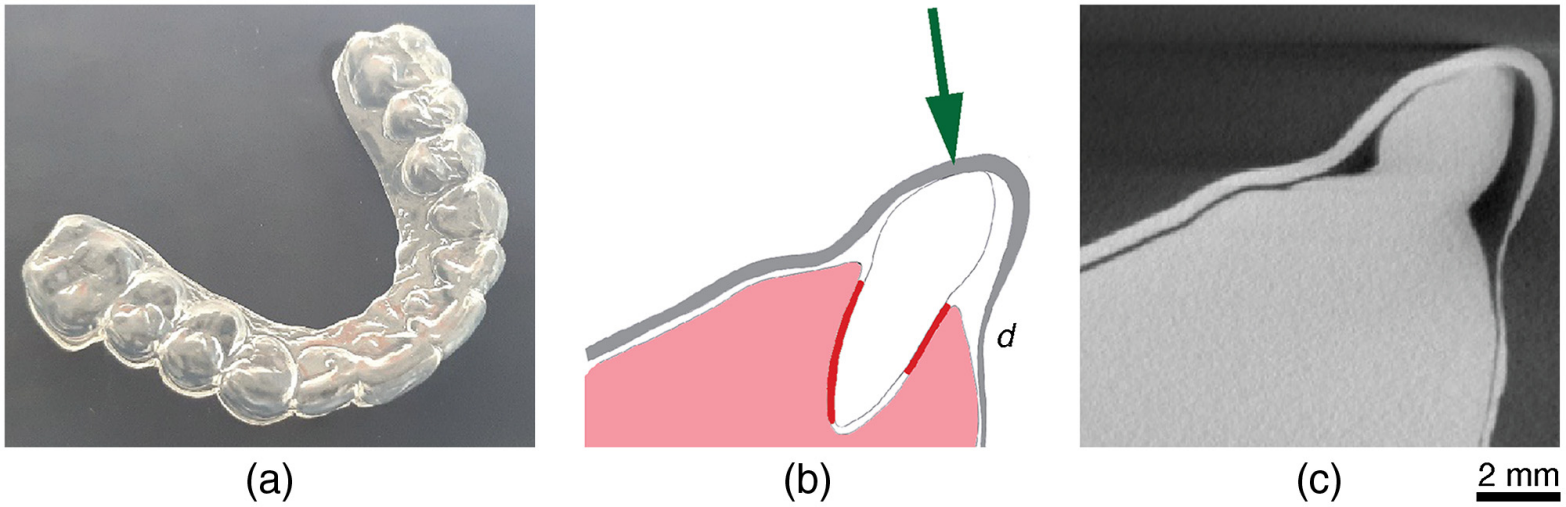
(a) An optically transparent aligner is a thermally processed polymer foil, which should fit the complex human dentition. (b) Following planning, the foil should generate force and torque on the teeth that need to be moved and rotated. Red-colored lines show the area of compression due to the force indicated by the green-colored arrow. The fit is critical, as gaps prevent force transmission, and the force amplitude correlates with the gray aligner thickness d. Therefore, the present tomography study aims at measuring local thicknesses and identifying gaps, clearly visible in (c) the tomographic slice of an aligner suboptimal for orthodontic treatments.

Aligners are used for treating mild-to-moderate and some complex malocclusions.^6^ In comparison with conventional orthodontic appliances, clear aligners are known to cause limited negative periodontal effects, as they can be removed before cleaning one’s teeth,^7^ and limited clinical emergencies, as they can be easily replaced if broken or lost.^8^ The rapid advancement of digital technologies in dentistry and in the field of orthodontics has led most aligner manufacturing companies to adopt a complete digital workflow. As intraoral optical scanners have become more accurate^9^ and easier to handle, more practitioners have been able to offer aligner treatments to their patients. Such a treatment normally starts with an intraoral scan of the upper and lower dental arch, and generated data are then processed using computer-aided design (CAD) to create virtual three-dimensional (3D) models. A treatment-planning program enables practitioners to virtually move the teeth in the desired position in steps that should range from 0.2 to 0.5 mm.^10^ For each step, a 3D model is created and printed using a stereolithographic technique (3D printer). These models are then used to create aligners with a thermoforming procedure using a polymer foil. Depending on the phase of the treatment, aligners of specific thicknesses can be used to modulate the force applied to the tooth.^11^ This choice is important, as excessive forces can cause hyalinization, bone necrosis, and external root resorption.^12^ Depending on the severity of the malocclusion, treatments last from 4 to 18 months.^8^ The success of aligner treatments depends on multiple factors such as the precision of the intraoral scanners and the 3D models,^6^ thickness and stiffness of the aligners,^13^^,^^14^ and fit on the dental arches (see Fig. 1).

Several polymeric materials are commercially available on the market. Many of them, however, release microplastics or even cytotoxic components.^15^ The recently introduced NaturAligner^®^ (Bottmedical AG, Basel, Switzerland), which comes in two thicknesses, namely, 550  μm, denoted NA.550, and 750  μm, denoted NA.750, is prepared from a bio-based material that avoids exposure to microplastics during treatment. Research on the interaction between microplastics and the human body is still ongoing, and long-term effects are thus only partly understood.^16^ Nevertheless, health hazards caused by immunological disturbances and chemical toxicity are currently being discussed, as well as their risks for cancer.^17^^,^^18^ Consequently, there are concerns about the effect of wearing aligners on a patient’s health, as they should be worn for 20 to 22 h a day for a total period of up to 18 months, depending on the treatment plan. The NaturAligner^®^ has a 25-μm-thin biopolymer coating based on cellulose acetate, which separates the force-generating polymer from the oral mucosa—thus preventing exposure.

Aligner thickness and fitting were evaluated by means of secondary electron microscopy,^19^^,^^20^ a two-dimensional (2D) technique with a large depth of focus. The current study is based on morphology measurements of the aligners using micro computed tomography, a method already successfully applied for investigation of the thickness and other geometric parameters of aligners.^21^^–^^24^ The 3D datasets of the present study were quantitatively analyzed to determine gap volume and local thickness changes as the result of the thermoforming process. These results support the engineer’s work in defining the parameters relevant for aligner production.

## 2 Methodology

### 2.1 Aligner Fabrication

The upper jaw of the subject was scanned using the intraoral scanner Medit i500 (Medit Corp., Seoul, South Korea). The subject showed noticeable abrasion on the incisors and canines due to bruxism. The generated data were then converted into the Standard Tessellation Language format and processed using CAD (Medit Link, Medit Corp., Seoul, South Korea) and triangle-mesh software (Meshmixer, Autodesk Inc., San Rafael). Artifacts caused by the scanning process were removed, and the edges of the model were smoothed to add a base to the models to ensure sufficient mechanical stability during the thermoforming process. Eight copies of the model were printed using the stereolithographic printer Formlabs Form3 (Formlabs, Somerville, Massachusetts) on the basis of the photopolymer resin Grey V4. All models were printed in 407 layers measuring 50  μm each, washed with isopropyl alcohol to remove residual liquid resin, and post-cured with ultraviolet light at a wavelength of 405 nm for a period of 15 min and a temperature of 60°C. This last step enhanced mechanical properties by terminating polymerization. A series of eight NaturAligners^®^ were thermoformed on the dental models with a pressure molding device (Biostar, Scheu-Dental, Iserlohn, Germany). It is noteworthy that the pressure molding device guarantees orientation and reproducibility of model placement for thermoforming.^19^ The process started by placing the foil in the holder and heating it with an infrared heater. A thermometer probe was placed right under the foil, and once the desired temperature was reached, the foil was positioned over the model and molded in the integrated pressure chamber by applying air pressure of 5.8 bar. After a cooling period of 1 min, the aligners were cut at the lower edge of the models but not dismounted. Each aligner had specific properties, as the two foil thicknesses of 550 and 750  μm were used. The four selected heating temperatures ranged from 112°C to 201°C.

### 2.2 Tomographic Imaging

NaturAligner^®^ specimens and the resin-based models exhibited similar local x-ray absorption values, so a simple intensity-based segmentation procedure was impossible. As the materials should not be modified by means of stains, this choice was a challenge of the study. Another challenge was the potential plastic deformation of the aligners as the result of removing them from the models. The scanning protocol was optimized to represent the aligner properly and to rule out any mechanical damage. For each aligner and model set, two sets of tomographic data were acquired: the aligner mounted on the model (model+aligner) and the model without an aligner (model). The models were fixed on a holder, allowing us to scan them in the same position during both scans. Subsequently, the corresponding model+aligner and model images were 3D registered. Tomographic data acquisition was performed with nanotom m (phoenix|x-ray, GE Sensing & Inspection Technologies GmbH, Wunstorf, Germany), equipped with a nanofocus tube for a maximal acceleration voltage of 180 kVp and the ability to generate power up to 15 W. In our case, we employed an acceleration voltage of 90 kVp and a beam current of 200  μA. The effective pixel length of the radiographs was set to 33  μm and resulted in (33  μm)^3^ voxels. The mean photon energy was increased by implementing a 0.5-mm-thick aluminum film behind the transmission target. A set of 2000 radiographs was taken along 360 deg with an exposure time of 2 seconds per projection. Scan duration was therefore ∼67  min.

### 2.3 Aligner Thickness Measurements

The bottom region of the model+aligner and the model image were rigidly registered with the open-source software Elastix.^25^^,^^26^ As the resin-based models and the aligners had similar local x-ray absorption values, aligner thickness was determined by subtracting the registered model from the model+aligner images. The resulting aligner mask was extracted via automatic thresholding using Otsu’s method, keeping the largest connected component and applying morphological image-closing. By extracting 2D centerline masks from all slices in the three orthogonal directions, and keeping centerline voxels that exist in more than one direction, the final center surfaces were determined. They were then used as reference points to measure thickness by calculating the distance to the nearest boundary point and multiplying it by a factor of two. This method was validated using the visualization program VGStudio Max 2.1 (Volume Graphics, Heidelberg, Germany). Two reference aligners, i.e., NA.550 processed at a temperature of 142°C and NA.750 processed at a temperature of 143°C, were chosen, and a total of 10 positions were randomly selected for semi-automatic thickness measurements. By applying an automated threshold and the function surface determination, defining the boundary between an object and its background based on their density, we were able to display the border of the aligner in an automated and a reproducible way. The distance between the borders of the aligner along a manually defined line segment was then determined with a digital measuring tool.

### 2.4 Gap Volume Determination

Gaps between aligner and model were extracted from the model+aligner image, based on thresholding the image with a fixed threshold, resulting in a binary mask. Three morphological image operations were employed to fill the gaps in each mask: first, dilation by a sphere of radius R, then filling holes, and finally erosion by a sphere of radius R. Gaps were defined by subtracting the binary masks from the filled masks. To determine the same region of interest, a plane 2 mm below the tooth-gingiva border was defined in the reference model NA.550 and processed at a temperature of 200°C. This plane was transferred to all model+aligner images by image registration, to remove image content below the plane as well as to seal the model+aligner image to support the hole-filling operation for the creation of the filled mask. This method was also validated using VGStudio Max 2.1 (Volume Graphics, Heidelberg, Germany) by manually segmenting all of the gaps in reference model NA.750 at a processing temperature of 176°C, as well as the gap of a region of interest, i.e., the buccal gap between the central incisors, for all models. To define this region of interest, all model+aligner images were aligned and cropped by predetermined coordinates. The region-growing method was used to semi-automatically segment the gaps, thereby determining whether a voxel should be included in the segmentation by defining a seed voxel and a suitable threshold.

## 3 Results

### 3.1 Validation of Automatic Measurement

The comparison of manual and automatic thickness measurements is shown in Table 1. With the semi-automatic method, we found a mean thickness of 165  μm for the NA.550 and 194  μm for the NA.750 aligners at five randomly selected positions each. Using the automatic measurement method, a mean thickness of 162  μm for the NA.550 aligner fabricated with a processing temperature of 142°C, and 168  μm for the NA.750 aligner prepared at a processing temperature of 143°C, was determined for the same positions. The mean absolute difference for the NA.550 aligner was found to be 15  μm, and for aligner NA.750 it amounted to 26  μm. It is worthy to note that the isotropic voxel size for all datasets was 33  μm. The consistency between the two measurement methods was quantified via the Pearson’s correlation coefficient ρ. They correlated with ρ=0.828 for the NA.550 aligner fabricated with a processing temperature of 142°C and with ρ=0.925 for the NA.750 aligner prepared at a processing temperature of 143°C.

**Table 1:** Comparison of automatic and manual thickness measurements of the NA.550 and NA.750 aligners fabricated at a processing temperature of 142°C and 143°C, respectively. The results were obtained at five arbitrarily selected positions, where the aligner was clearly separated from the model surface.

	NA.550				
Automatic (μm)	162	187	114	187	162
Manual (μm)	181	166	128	176	172
Difference (μm)	-19	21	14	11	−10
	NA.750				
Automatic (μm)	187	219	114	187	132
Manual (μm)	203	237	148	195	186
Difference (μm)	−16	−18	−34	−8	−54

The comparison of the automatic and manual segmentations of gaps also showed consistency (see Table 2). Using a fixed gray-value threshold of 150 and automatic segmentation, a total gap volume of $24.6\;mm^{3}$ was determined for a selected aligner; this volume was spread over approximately four dozen gaps along 14 teeth of the selected aligner NA.750 processed at a temperature of 176°C. Manual segmentation of the same aligner resulted in a total volume of $29.4\;mm^{3}$. The difference between the two methods was found to be $4.8\;mm^{3}$ (see the last column of Table 2).

**Table 2:** Automatic determination of the volume of one selected gap (ROI), the buccal gap between the central incisors, and total gap volume (all gaps) in comparison to manual segmentation.

Aligner	ROI								All gaps
	NA.550				NA.750				NA.750
Process temperature (°C)	112	142	173	200	112	143	176	201	176
Automatic (${\mathrm{mm}}^{3}$)	24.8	6.9	1.7	2.2	16.7	2.2	2.0	1.2	24.6
Manual (${\mathrm{mm}}^{3}$)	25.4	7.0	1.0	1.3	16.4	2.9	1.7	1.3	29.4
Difference (${\mathrm{mm}}^{3}$)	−0.6	−0.1	0.7	0.9	0.3	−0.7	0.3	0.1	−4.8

The results from segmenting the buccal gap between the central incisors for all aligners are shown in the columns headed by ROI (see Table 2). The mean gap volume for automatic segmentation totaled $8.9\;mm^{3}$ for the NA.550 and $5.5\;mm^{3}$ for the thicker NA.750 aligners. Also employing the fixed gray-value threshold of 150 as a starting value for the manual segmentation, we found mean gap volumes of $8.7\;mm^{3}$ for the NA.550 and $5.6\;mm^{3}$ for the NA.750 aligners. The mean absolute difference between automatic and manual procedures was $0.6\;mm^{3}$ for the NA.550 and $0.3\;mm^{3}$ for the NA.750 aligners. The related Pearson’s correlation coefficients were 1 and 0.998, respectively. Taking the correlation coefficients and mean absolute difference values into consideration, we validated the reliability of the automatic gap volume and aligner thickness measurement methods.

### 3.2 Aligner Thickness Distribution

The results of the local aligner thickness measurements are given in Table 3. Using the automatic measurement method, the following median aligner thicknesses were determined for all center-surface voxels. For the NA.550 aligners, the median thickness was 361  μm using a processing temperature of 112°C, 337  μm using a processing temperature of 142°C, 330  μm using a processing temperature of 173°C, and 330  μm using a processing temperature of 200°C. Concerning the NA.750 aligners, we found 462  μm using a processing temperature of 112°C, 428  μm using a processing temperature of 143°C, 443  μm using a processing temperature of 176°C, and 448  μm using a processing temperature of 201°C. The overall thickness of the aligners was therefore almost constant in the temperature range studied, but it was substantially smaller than the thickness of the planar foils employed as the starting material in the thermoforming process.

**Table 3:** Summary statistics of thickness distribution values for the entire datasets and for 100 randomly selected voxels on the center-surface.

	NA.550				NA.750			
Process temperature (°C)	112	142	173	200	112	143	176	201
All data used								
Minimum (μm)	66	66	66	66	66	66	66	66
Median (μm)	361	337	330	330	462	428	443	448
Mean (μm)	336	330	329	320	440	427	429	430
Maximum (μm)	594	579	676	689	761	888	919	956
Standard deviation	99	74	73	79	87	96	114	154
100 data points								
Minimum (μm)	66	162	93	66	187	148	66	66
Median (μm)	373	337	337	330	462	438	435	485
Mean (μm)	338	339	338	321	447	436	427	455
Maximum (μm)	485	480	528	568	583	590	660	717
Standard deviation	97	62	64	97	81	82	113	152

**Fig. 2:**
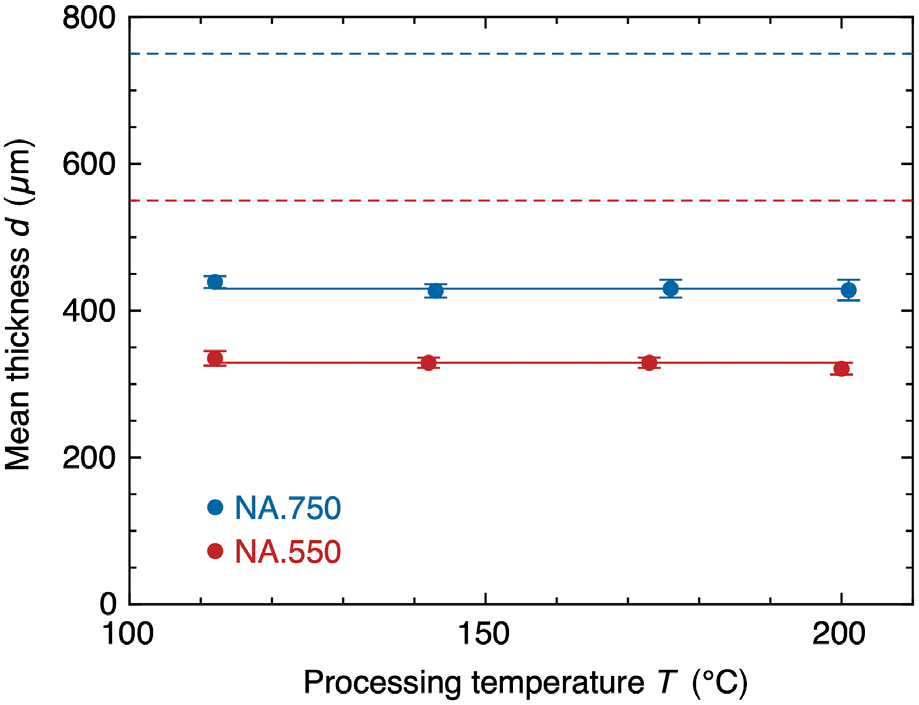
Thickness distribution of the mean when sampling 100 center-surface voxels 100 times, with solid lines showing the average value. The dashed lines show the thickness of the foils before thermoforming.

As the number of center-surface voxels averaged 3 million per aligner, we provide a simplified representation of the results in Fig. 2. The diagram shows the mean thickness when sampling 100 random points 100 times. The values were relatively stable to resampling, thus allowing for a reasonable approximation. The NA.550 foils were on average 334-μm-thick, while the NA.750 foils resulted in a thickness of 441  μm after the thermoforming process. These results demonstrate a shrinkage of 60.7% and 58.8% for the NA.550 and NA.750 aligners, respectively.

**Fig. 3:**
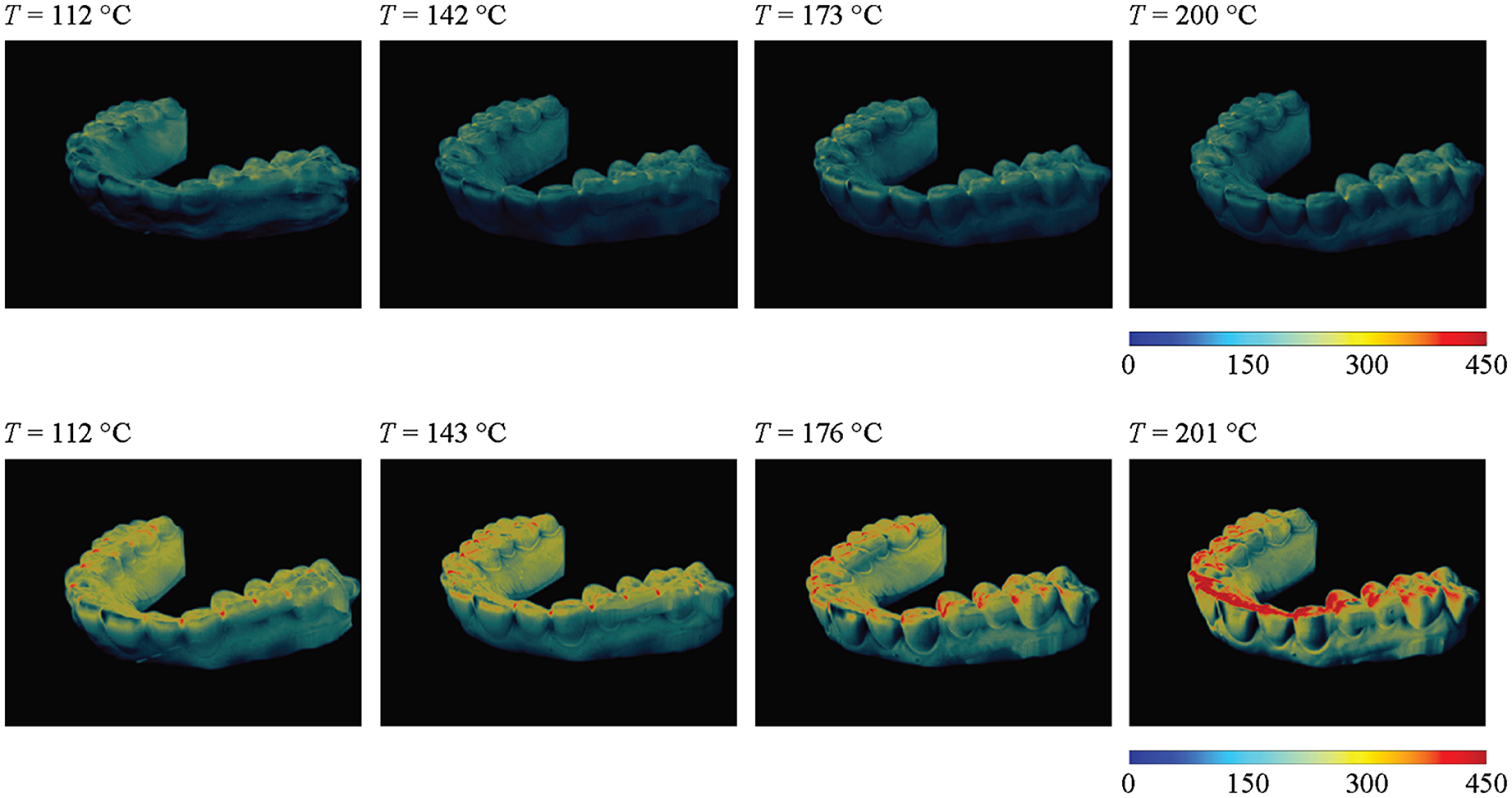
Color-coded aligner thickness distribution for the NA.550 aligners, row 1, and NA.750, row 2. Color bars show thickness in μm. Processing temperatures during thermoforming are also provided.

Although the mean and median thickness values were stable, there was a difference in thickness distribution after the thermoforming process. The maximum intensity projections, represented in Fig. 3, show the thickness distribution of the mounted aligners in 3D space. The NA.550 aligners were generally thicker on the occlusal surfaces of molars and premolars but thinner around the incisors and buccal as well as oral surfaces. Although the NA.550 produced at a processing temperature of 112°C was slightly thicker on said zones when compared to other NA.550 aligners, thickness distribution was fairly even across the selected temperature range. This behavior indicates that the NA.550 aligner was hardly affected by changes in selected processing temperatures—and thus stable in terms of the thermoforming process. The NA.750 aligners, however, showed noticeable differences when exposed to rising process temperatures. Visible changes occurred between 143°C and 173°C and were accentuated using processing temperatures of 201°C. Similar to its thinner counterpart, NA.750 foils were thicker on the occlusal surfaces of molars and premolars, but changes became clearer in line with rising temperatures, as the foils became thicker on the palatal surface of all front teeth as well as on the palate itself. Therefore, we could conclude that the selected temperature of thermoforming process has an impact on the thickness distribution of NA.750 foils, contrary to the NA.550 ones.

### 3.3 Gap Volume

**Fig. 4:**
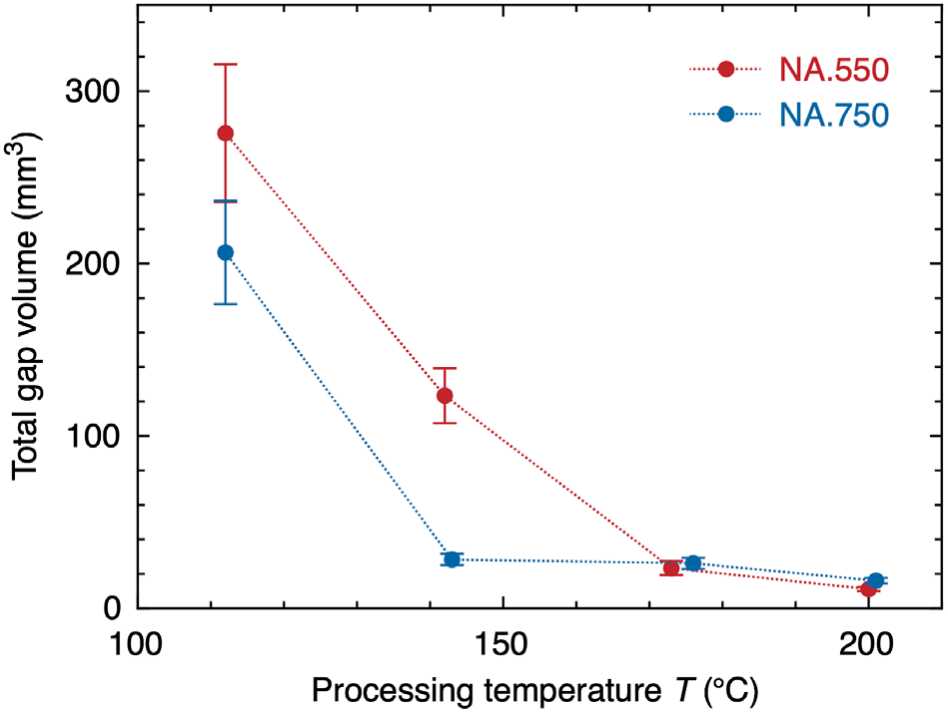
Total volume statistics for all gaps derived from the automatic method. The error bars were determined using the three threshold values 140, 150, and 160. The dotted lines, which connect the data, should just guide the eyes.

The results of the gap segmentations are shown in Fig. 4. Selecting the proper threshold was critical, as taking inappropriate values resulted in inaccurate segmentations. As the foils completely thinned out in some areas, there was no optimal threshold for segmenting all gaps perfectly; therefore, using the automatic segmentation method as mentioned above, we measured the gaps of each aligner with three thresholds, namely, 140, 150, and 160, and calculated the average for each processing temperature and aligner thickness. Thermoforming the aligners on the corresponding models resulted in the following mean gap volumes: $268.9\;{\mathrm{mm}}^{3}$ for the processing temperature of 112°C, $115.3\;mm^{3}$ for the processing temperature of 142°C, $21.9\;mm^{3}$ for the processing temperature of 173°C, and $11.2\;mm^{3}$ for the processing temperature of 200°C using the NA.550 foils. Using the thicker NA.750 foils, we found $191.1\;mm^{3}$ for the processing temperature of 112°C, $26.3\;mm^{3}$ for the processing temperature of 143°C, $24.8\;mm^{3}$ for the processing temperature of 176°C, and $15.72\;mm^{3}$ for the processing temperature of 201°C. Using the thermoforming temperature of 112°C, the NA.750 foil had a mean gap volume about 30% smaller than the one for NA.550 (see Fig. 4). Increasing the temperature in steps of about 30 K, one recognizes a reduction in the total gap volume for both foil thicknesses. Data for the NA.550 displayed in Fig. 4 diagram correspond to percentual volume decreases to 57%, 35%, and 4%, respectively, per increasing process temperature step. The aligners made with the 750-μm-thick foil decreased to 86%, 1%, and 5%, respectively, for the individual 30 K process temperature steps.

**Fig. 5:**
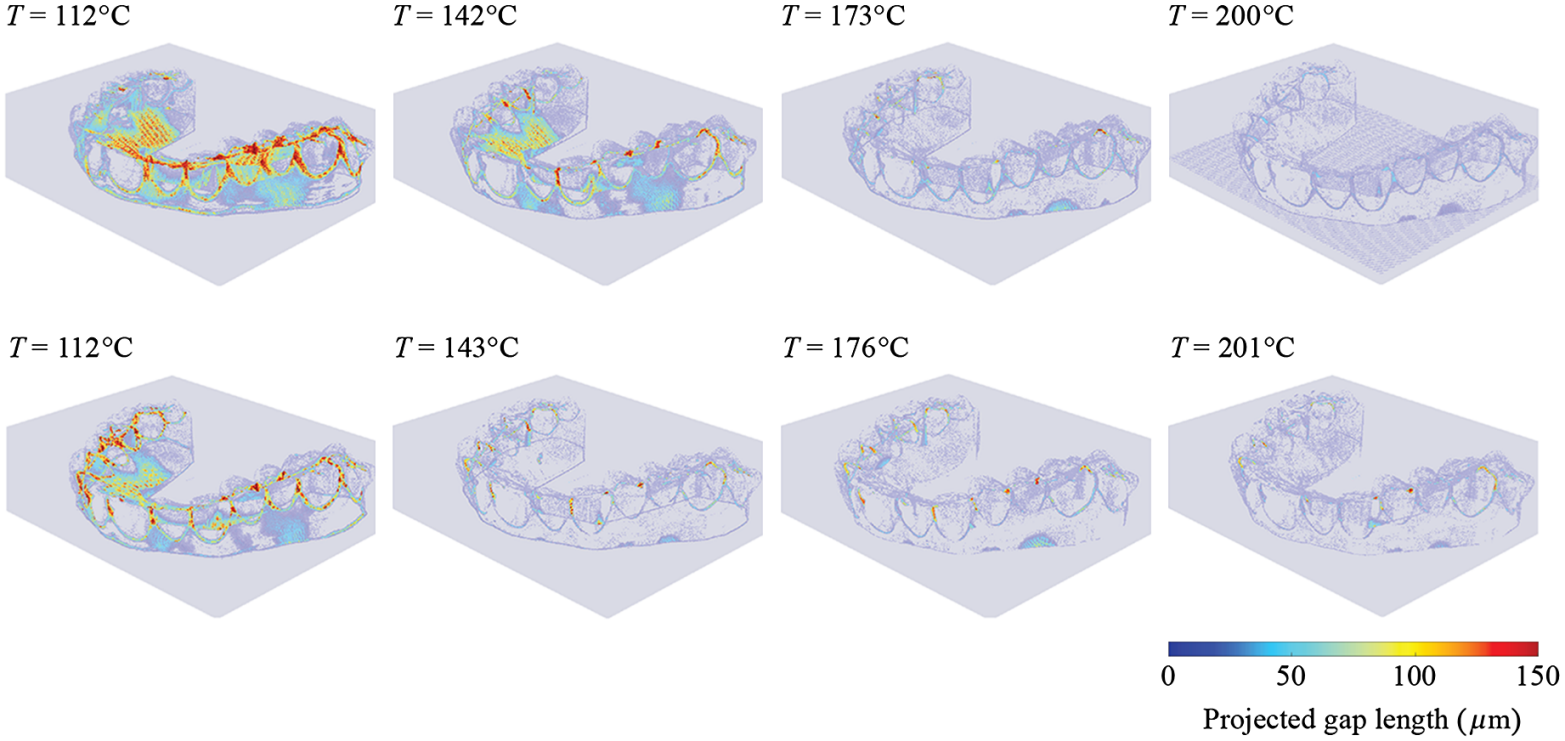
Rendering of the projected gap lengths for the NA.550 aligners (top row) and NA.750 aligners (bottom row). The selected processing temperatures are indicated.

The rendering of Fig. 5 shows the gap size distribution for the selected process temperatures. It is distinctly visible that the areas where gaps form throughout the selected temperature range are between the teeth and at the tooth-gum border. At low processing temperatures, areas around the palate also show larger gaps. The buccal and palatal tooth surfaces show no visible gaps at higher processing temperatures. This experimental result is important, as those surfaces are central to orthodontic tooth movements. We can therefore conclude that aligners made with NA.750 foils have smaller gaps at the same process temperature. The rise in temperature affects the gap volume as expected. Although the difference becomes negligible at higher processing temperatures, where the remaining gaps seem to be inevitable, due to the morphology of teeth.

## 4 Discussion

### 4.1 Clinical Perspective

In patients undergoing orthodontic treatment using aligners, the vast majority of orthodontic tooth movement, during any 2-week aligner prescription cycle, occurs during the first week of the cycle.^27^ This varied tooth movement is due to the type of material, thickness, and gap volume. The most common approaches for aligner treatment are: (i) implementing smaller tooth movements in each setup, which results in more treatment steps, or (ii) using thinner and less stiff aligners while maintaining the tooth movement recommendation of 0.2 to 0.5 mm. In our study, however, we explored an alternative approach based on the thickness of the foils to negate factors such as gap volumes and intensity of forces. We found that the use of both foil thicknesses might be beneficial in achieving the best outcomes in an individual patient plan. In the initial phase of an orthodontic treatment, mild forces should be applied, to avoid any adverse effects on the teeth and the surrounding tissue, by choosing the right aligner stiffness and also taking gap volumes as well as their position into consideration. Choosing an aligner that does not apply too much force on teeth, therefore, seems central to this matter. While comparing thinner and thicker aligners, a study found that the latter will deliver significantly greater forces.^28^ The authors concluded that there was a strong correlation between foil thickness and delivered forces, in that thinner foils are more flexible and therefore better suited for the initial phase of an aligner treatment. Once this phase is over, higher orthodontic forces can be applied using thicker foils. Practitioners should be aware that the thickness distribution of NA.750 is altered when processing temperatures above 170°C are employed. They can use this information to take advantage, as higher forces can be applied vertically on upper molars and incisors, thus making intrusions and protrusions easier. Intrusive forces from the aligner are critical;^29^ and root resorption is a common adverse effect of orthodontic tooth movement and can occur in all types of tooth movements, especially intrusion and uncontrolled tipping, while most corrections require a combination of these movements.^30^

Bruxism caused the subject’s abrasion of incisors and canines. Patients affected by this oral parafunction grind and press their teeth with elevated forces for a prolonged period of time. The use of an aligner reduces tooth abrasion, but generates microplastics within the oral cavity, which eventually reaches the blood stream. Practitioners should take this danger into consideration when choosing the aligner system.

### 4.2 Aligner Fitting Precision

A study investigating aligner fitting accuracy with laboratory-based micro computed tomography found that six commercially available aligners had a gap volume ranging between 107 and $402\;mm^{3}$ in the studied temperature range but with a region of interest that did not include the entirety of teeth.^22^ In our study, a plane 2 mm under the lowest tooth-gum border was defined, allowing us to broaden the region of interest to all teeth. Even with a larger region of interest, the NaturAligner^®^ gaps were at least similar to or substantially smaller than the six aligner brands included in the study mentioned previously. Another important step regarding aligner fitting accuracy during the manufacturing process could be the dismounting of the aligner from the models. Using high forces can lead to plastic deformation—and thus to permanently damaged aligners. This in turn could affect force delivery, as they will no longer fit properly on the dental arch.

### 4.3 Mechanical Properties

The oral cavity creates a particular environment, as it is close to body temperature, humid, and subject to mechanical as well as chemical stress caused by teeth, saliva, food, and beverages. The protocols of many commercially available aligners state that they should be worn 20 to 22 h a day and changed after 1 to 2 weeks. Each aligner is therefore exposed to conditions in the oral cavity for about 140 to 310 h. An ideal aligner should be able to exert a constant and an equal force over this period of time. Therefore, it seems important that mechanical properties, including stiffness, hardness, and elasticity, do not significantly change due to intraoral conditions and regular usage. Although it is recommended to study the mechanical properties of thermoplastic foils after the thermoforming process,^31^ preliminary results show that foils are subject to a substantial stress decrease in the hours following the application of an initial force.^32^ Cyclic forces, which emulate forces occurring during chewing and swallowing movements, also appear to have an impact on the delivered forces, as mechanical properties are altered.^33^ Changes include decreased wear resistance, increased brittleness, and stiffness as well as deformation.^34^^,^^35^ Also, aligners need to be inserted and removed several times daily before and after meals, as well as for oral hygiene. Study conclusions on the effects of removal frequency on deformation, and thus force delivery, are not unanimous.^33^^,^^36^ Although the clinical relevance of the change in mechanical properties has to be demonstrated, as most studies have an *in vitro* design, possible differences in force delivery during intraoral use must be taken into consideration when choosing an aligner brand. Our study also falls into this category, as all measurements were made on a resin cast. Building on the insights gained in this study, further research with adapted study designs could be conducted to measure the effect of the *in vivo* usage of NaturAligner^®^ and the possible consequences on the predictability of an orthodontic treatment.

### 4.4 Attachments

Certain types of orthodontic movements, such as extrusion, mesio-distal root tip, and rotation of lateral incisors, canines or first premolars, are poorly predictable with aligners. In those cases, auxiliaries called “attachments” can help make a treatment outcome more predictable. They are made out of composite, fixed on teeth, and allow aligners to exert torque and tooth rotation, which would be impossible otherwise.^6^ Dental composites are a type of material primarily used for dental fillings, and their mechanical and optical properties depend on their composition. Examples with a high percentage of filler are called packable composite and with a low percentage flowable composite. Considering the fact that an aligner needs to fit the dental arch precisely, it seems evident that it should also do so for attachments. Even though the shape of an attachment and the type of composite seems to play a central role in retention—and thus force and torque delivery—aligners generally fit attachments quite well.^37^ Therefore, we can hypothesize that the NaturAligner^®^ remains similar to other aligners. Nevertheless, further investigation should be conducted to assess the fitting precision of NaturAligner^®^ to such attachments.

### 4.5 Optical Properties

The visibility of orthodontic devices can influence the way a person is perceived and may influence a patient’s choice of appliance.^2^ An aligner’s transparency appears to be a key feature for patients opting for an aligner treatment. However, the optical properties of aligners can change due to pigments from food and beverages.^38^^,^^39^ As the outcome of an aligner treatment is heavily influenced by the numbers of hours a patient wears them,^3^ it seems important that patients do not feel any social discomfort, as it might influence their compliance. For this reason, the stability of optical properties of NaturAligners^®^ should be taken into consideration for further studies.

## 5 Conclusion

Advanced laboratory-based hard x-ray tomography is a reliable method to measure aligner gap volume and thickness distribution. The segmentation procedure is challenging, though, since the x-ray absorption values of the models and aligners are similar. The proposed procedure can be used for any other aligner and similar device. NaturAligner^®^ fits dental arches with high levels of precision, and the selected foil thicknesses make differential orthodontic tooth movements possible. The initial phase of orthodontic treatment can be better accomplished with NA.550, while the later phase of tooth movement is viable with NA.750.
